# Synchronous invasive ductal carcinoma and intravascular large B-cell lymphoma of the breast: a case report and review of the literature

**DOI:** 10.1186/1477-7819-12-88

**Published:** 2014-04-08

**Authors:** Christopher Wei Guang Ho, Sangeeta Mantoo, Chin Hong Lim, Chow Yin Wong

**Affiliations:** 1 Department of General Surgery, Singapore General Hospital, Outram Road, Singapore 169608, Singapore; 2 Department of Pathology, Singapore General Hospital, Outram Road, Singapore 169608, Singapore

**Keywords:** Intravascular large B-cell lymphoma, Synchronous tumour, Primary breast lymphoma, Breast cancer, Invasive ductal carcinoma

## Abstract

Primary breast lymphomas (PBLs) represent less than 1% of all breast malignancies. Intravascular large B-cell lymphoma (ILBCL) is a rare, aggressive form of extranodal lymphoma. Breast involvement has only been described in the literature once previously. ILBCL is characterized by the proliferation of tumour cells within the lumen of small vessels of involved organs, resulting in their eventual occlusion. Clinical features are often vague, diagnosis is difficult and delayed, and prognosis is usually poor. We report the first ever case of synchronous ILBCL and invasive ductal carcinoma (IDC) of the breast in a patient presenting with pyrexia of unknown origin and altered mental status who underwent modified radical mastectomy and subsequent chemotherapy, and review the literature regarding intravascular large B-cell lymphoma, PBLs and synchronous carcinomas and lymphomas of the breast.

## Background

The breast is one of the least common sites for malignant extranodal lymphomas. Primary breast lymphomas (PBLs) constitute less than 1% of all breast neoplasms, and are usually non-Hodgkin’s lymphomas. A wide variety of histological subtypes have been reported, and it is thought that patients with PBLs have a stage-for-stage prognosis similar to other patients with lymphomas of the same histology that present at other sites [1]. PBLs usually present as a painless, solitary mass, often with associated ipsilateral axillary lymphadenopathy. Multiple masses and diffuse breast enlargement (unilateral or bilateral) have also been reported. Skin retraction, erythema, peau d’orange and nipple involvement are rare. The most frequent mammographic appearance is a well-circumscribed uncalcified mass. Other radiographic features include amorphous or spiculated uncalcified masses and diffusely increased parenchymal density. Ultrasound usually reveals hypoechoic lesions with well-defined borders that lack significant posterior enhancement or acoustic shadowing that may be confused with a benign cyst [2].

Intravascular large B-cell lymphoma (ILBCL) is a rare, high-grade extranodal diffuse B-cell lymphoma that has only been described in the breast once previously in a patient who had bilateral symmetrical breast engorgement with thickened, peau d’orange skin [3]. The median age at diagnosis is 67 years and it has a male:female sex distribution of 1.3:1. ILBCL may affect virtually any organ and is characterized by the clonal proliferation of malignant lymphocytes within the lumen of small vessels, resulting in their consequent blockage. Parenchyma, lymph nodes and the reticuloendothelial system are relatively spared [4]. In contrast to other lymphomas that present with lymphadenopathy and tumour mass, ILBCLs present heterogeneously depending on the organ affected. Common clinical features include pyrexia of unknown origin, generalized fatigue, cutaneous lesions (including rashes, plaques, nodules, ulcers and hyperpigmentation) and neurological changes (including altered consciousness, sensorimotor deficits, seizures, paresis and dementia). Gastrointestinal symptoms, anaemia, thrombocytopenia, oedema and dyspnoea have also been reported. Due to its wide-ranging, non-specific signs and symptoms, the diagnosis of ILBCL is often difficult and treatment consequently delayed. Combined with its aggressive nature, disseminated disease is often present when ILBCL is ultimately recognized, resulting in a dismal prognosis [4].

We describe the first case of synchronous ILBCL and invasive ductal carcinoma (IDC) of the breast, and illustrate the diagnostic pitfalls and therapeutic dilemmas commonly encountered by clinicians when faced with ILBCL and synchronous tumours.

## Case presentation

A 75-year-old Malay woman with a background of diabetes mellitus, hypertension, hyperlipidaemia and endometrial carcinoma who had undergone curative resection and was now in remission presented with a 6-month history of flu-like symptoms and intermittent swinging pyrexia associated with chills and rigors. The patient’s family had also noticed that she was becoming increasingly confused and drowsy.

The patient had no gastrointestinal, genitourinary or respiratory symptoms, and did not have signs of meningitis. Physical examination was normal. Extensive infective, malignancy and autoimmune screens did not yield any positive results. Repeated computed tomography (CT) and magnetic resonance imaging (MRI) of the brain were unremarkable. Bone marrow biopsy revealed a hypercellular marrow without any evidence of haematolymphoid malignancy or metastasis and was sent for karyotyping. Bone marrow lymphocytes showed normal morphology and featured a diffuse interstitial infiltrate comprising CD3 positive T-cells with fewer CD20 positive B-cells.

**Figure 1:**
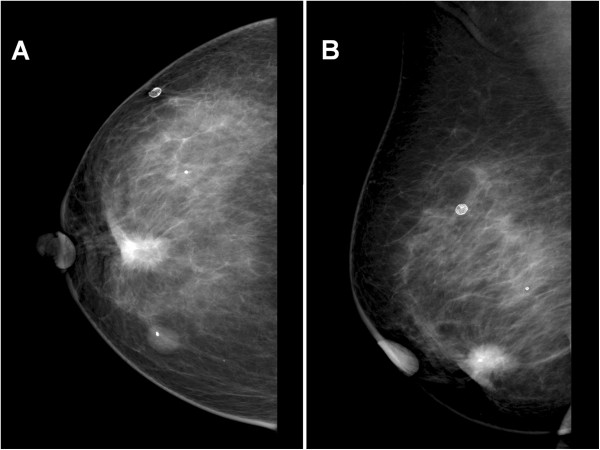
**Mammogram. (A)** Craniocaudal and **(B)** mediolateral oblique views of the patient’s right breast. There is a 19 mm spiculated mass with associated architectural distortion in the lower central breast highly suspicious for malignancy. There is also a partially calcified fibroadenoma in the lower inner quadrant and a few calcified oil cysts in the upper outer quadrant.

**Figure 2:**
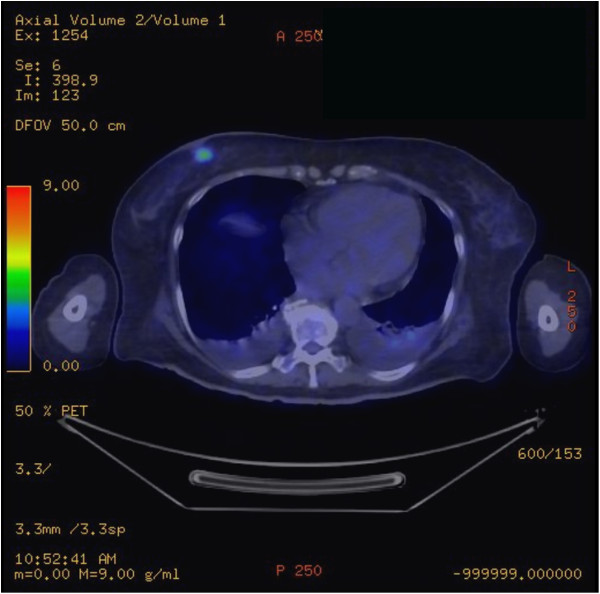
**PET-CT.** A 1.8 × 1.4 cm spiculated mildly FDG-avid right breast nodule just below the level of the nipple is demonstrated (maximum standardized uptake value 3.9). FDG, fluorodeoxyglucose; PET-CT, positron emission tomography-computed tomography.

CT of the thorax, abdomen and pelvis was unremarkable except for a 16 mm lesion in the right inferior breast. Mammography confirmed an irregular 12 × 11 × 9 mm spiculated mass in the 6 o’clock position with ill-defined margins (Figure 1). Fine needle aspiration cytology was suggestive of malignancy, and positron emission tomography (PET)-CT showed a hypermetabolic right breast nodule suspicious for primary breast tumour (Figure 2). No other fluorodeoxyglucose (FDG)-avid lesions were noted.

As extensive investigations had failed to identify the reason for the patient’s symptoms, the presumptive diagnosis was that of a breast carcinoma-related paraneoplastic syndrome causing fever and confusion. A modified radical mastectomy with level II axillary clearance was performed, and the fever resolved immediately after surgery. The patient also became more alert, albeit only for the first 2 postoperative days before becoming drowsy again.

**Figure 3:**
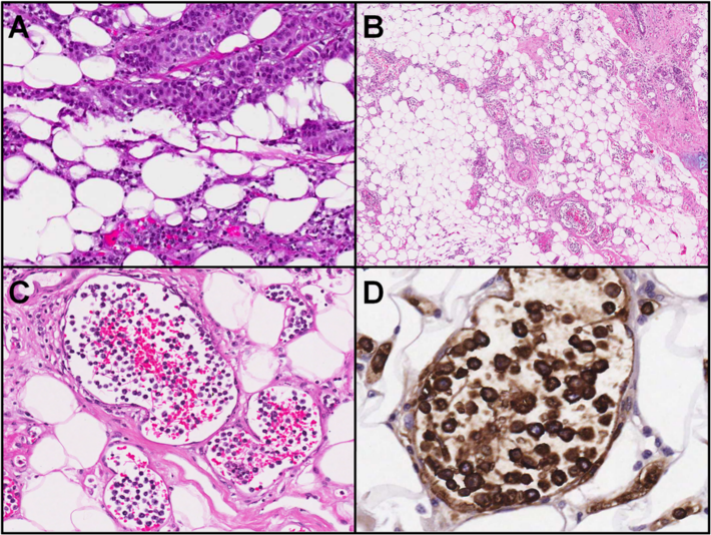
**Histological analysis. (A)** IDC (top right) with ILBCL (bottom centre) 130× magnification. **(B)** ILBCL in the surrounding breast tissue away from the IDC. Most of the blood vessels show darkly stained atypical lymphoid cells within their lumen 40× magnification. **(C)** Atypical lymphoid cells within the blood vessels 200× magnification. **(D)** CD20 reactive atypical large cells within the blood vessels 400× magnification. IDC, invasive ductal carcinoma; ILBCL, intravascular large B-cell lymphoma.

Histological analysis confirmed a 20 mm IDC (grade I, oestrogen receptor (ER) and progesterone receptor (PR) positive, human epidermal growth factor receptor 2 (HER2) negative) with minimal ductal carcinoma *in situ*. In addition, the small- and medium-sized vessels surrounding the focus of IDC and the breast tissue revealed large atypical intravascular cells (Figure 3A,B,C). These atypical cells were CD20 positive B-lymphocytes with small to moderate amounts of cytoplasm and round to irregular, vesicular and nucleolated nuclei with discernible mitoses (Figure 3D). These large lymphoid cells were strongly and diffusely reactive to MUM-1, and showed weak positivity for CD5, BCL6 and CD10. No expression was seen with cyclin D1, CD23 and CD3. MIB1 proliferative index was more than 90%. Bone marrow karyotyping also revealed that 2 out of 20 metaphases analyzed had complex structural abnormalities, loss of X chromosome and trisomy 18. Thus, a unique diagnosis of synchronous IDC and non-germinal centre type ILBCL was made.

The patient was started on the rituximab, cyclophosphamide, hydroxydaunorubicin, Oncovin (vincristine) and prednisolone (R-CHOP) chemotherapy regimen which led to a sustained improvement in her mental state. Unfortunately, the subsequent tumour lysis resulted in acute renal failure requiring haemodialysis. Finally, the patient developed neutropenic sepsis, and passed on 2 weeks after commencing chemotherapy.

## Discussion

This case represented a significant diagnostic challenge because of the patient’s non-specific clinical features, the presence of a confounding synchronous IDC and the rarity of ILBCL, especially in the breast. Similar to the previous reported case of ILBCL involving the breast [3], this patient also satisfied the diagnostic criteria for primary breast lymphomas, but did not have any signs or symptoms localizing to the breast. One of the differentials entertained was a paraneoplastic syndrome, which results from tumour secretion of substances that mimic the effects of endogenous hormones and cytokines, or from the production of antitumour antibodies that cross-react with normal cells. Paraneoplastic syndromes involving the breast are rare, and it is estimated that only 36% of such patients have identifiable paraneoplastic antibodies [5]. Fever is one of the most common features, and is thought to be cytokine-mediated. In addition, specific onconeural autoantibodies such as anti-Ri, anti-Ro and anti-Yo, which may form in response to the breast neoplasm, may also target tissue antigens of the central and peripheral nervous system [5,6]. However, in contrast to this case, the neurological manifestations described were motor, sensory and visual with no impairment of consciousness.

Given its rarity and protean manifestations, the diagnosis of ILBCL is one of considerable difficulty. Nevertheless, ILBCL often presents with pyrexia of unknown origin, as well as cutaneous and neurological features [4]. Neuroradiological signs, however, are present in only half of patients with neurological symptoms; CT is frequently normal in patients with neurological manifestations of ILBCL and is therefore generally considered non-diagnostic, and MRI is often normal or shows non-specific hyperintense white matter lesions suggestive of small vessel ischaemic disease or demyelination [7]. As illustrated in this case, PET-CT may also fail to demonstrate cerebral localization of ILBCL. Such false negatives have also been reported in other studies in patients with neurological symptoms [4,8]. Bearing this in mind, a low threshold for biopsy of suspicious lesions should be maintained in the appropriate clinical context, as histological diagnosis is paramount to the timely and appropriate treatment vital to improving patient outcome. The classic appearance and immunophenotype of ILBCL is that of large malignant CD20, CD19, CD10, CD5 and CD79a positive B-lymphocytes filling small vascular lumens. In addition, chromosomal analysis may reveal abnormalities in 1p and trisomy 18 [4,9].

Numerous theories attempt to explain the pathogenesis of synchronous carcinoma and lymphoma of the breast. Patients with lymphoma are known to be chronically immunosuppressed, which may predispose to the development of a second malignancy [10]. It has also been suggested that the antigenic stimulation from an as yet undefined breast carcinoma antigen may drive the development of a mucosa-associated lymphoid tissue (MALT) lymphoma, analogous to the effect of chronic *Helicobacter pylori* infection on the pathogenesis of gastric MALT lymphomas [11]. Another postulated mechanism is that both tumours share the same aetiological factors, with mutation in the ataxia telangiectasia mutated (ATM) tumour suppressor gene [12], as well as infection with the Epstein-Barr virus (EBV) [13] and mouse mammary tumour virus (MMTV) [14] all currently implicated.

Mastectomy has traditionally been considered the gold standard treatment for PBLs, however, this has now shown to offer no survival benefit or protection from recurrence [2]. Chemotherapy, typically an anthracycline-based regime with or without rituximab has emerged as the mainstay of treatment, and can be combined with radiotherapy in patients with intermediate or high grade lymphomas [1,2,15]. There are no randomized controlled trials comparing treatment for patients with ILBCL, however, as dissemination is common at diagnosis, aggressive systemic chemotherapy is usually warranted. Methotrexate, either intrathecal or high-dose systemic, should be incorporated if central nervous system involvement is present [16]. The addition of rituximab has been shown to significantly improve clinical outcomes [4,17], and autologous stem cell transplantation has been shown to be effective in young patients with good performance status [17]. Because of the rarity of synchronous carcinoma and lymphoma of the breast, there is no consensus on treatment in such cases and it remains uncertain if such synchronous tumours should be viewed as two distinct clinical entities with corresponding separate treatments, or as a single disease with therapy that encompasses both tumour types.

This unique synchronous tumour comprising of an IDC and ILBCL in a patient presenting with pyrexia of unknown origin and altered mental status highlights the difficulties in diagnosing ILBCLs as well as synchronous tumours in general. Although the optimal management for such synchronous tumours is still a matter for further studies, adequate histological assessment is vital to ensure appropriate treatment. Finally, additional investigation into the pathogenesis of synchronous carcinoma and lymphoma of the breast may lead to novel preventative and therapeutic strategies for such synchronous tumours as well as for its individual constituent neoplasms.

## Conclusion

We report the first case of synchronous carcinoma and ILBCL of the breast. ILBCL is rare, and has only been described in the breast once previously. Its clinical spectrum is broad, and a high degree of suspicion is required to seek early biopsy to establish histological diagnosis. Timely, aggressive, systemic chemotherapy has been shown to significantly improve outcomes. The development and treatment of synchronous breast carcinomas and lymphomas remains uncertain, and further studies should be encouraged.

## Consent

Written informed consent was obtained from the patient for publication of this case report. A copy of the written consent is available for review by the Editor-in-Chief of this journal.

## Abbreviations

ATM: Ataxia telangiectasia mutated; CT: Computed tomography; EBV: Epstein-Barr virus; ER: Oestrogen receptor; FDG: Fluorodeoxyglucose; HER2: Human epidermal growth factor receptor 2; IDC: Invasive ductal carcinoma; ILBCL: Intravascular large B-cell lymphoma; MALT: Mucosa-associated lymphoid tissue; MMTV: Mouse mammary tumour virus; MRI: Magnetic resonance imaging; PBL: Primary breast lymphoma; PET: Positron emission tomography; PR: Progesterone receptor; R-CHOP: Rituximab cyclophosphamide, hydroxydaunorubicin, Oncovin, prednisolone.

## Competing interests

The authors declare that they have no competing interests.

## Authors’ contributions

CWGH, CHL and CYW treated the patient and conceived the idea. SM performed the pathological analysis and provided the histology slides. CWGH performed the literature search and wrote the manuscript. CYW reviewed and revised the manuscript. All authors read and approved the final manuscript.
